# Consensus on the Application of Lung Ultrasound in Pneumonia and Bronchiolitis in Children

**DOI:** 10.3390/diagnostics10110935

**Published:** 2020-11-11

**Authors:** Joanna Jaworska, Anna Komorowska-Piotrowska, Andrzej Pomiećko, Jakub Wiśniewski, Mariusz Woźniak, Błażej Littwin, Magdalena Kryger, Piotr Kwaśniewicz, Józef Szczyrski, Katarzyna Kulińska-Szukalska, Natalia Buda, Zbigniew Doniec, Wojciech Kosiak

**Affiliations:** 1 Cystic Fibrosis Department, Institute of Mother and Child, 01-211 Warsaw, Poland; joanna.jaworska@imid.med.pl; 2 Pediatric Pulmonology and Allergy Department, Medical University of Warsaw, 02-091 Warsaw, Poland; anna.komorowska-piotrowska@wum.edu.pl; 3 Clinic of Pediatrics, Hematology and Oncology, University Clinical Center, 80-210 Gdansk, Poland; apomiecko1@gmail.com (A.P.); wisniewski@gumed.edu.pl (J.W.); blittwin88@gmail.com (B.L.); jozefszczyrski@gmail.com (J.S.); magdalena.kryger@gmail.com (M.K.); 4 Department of Pulmonology, Institute of Tuberculosis and Lung Diseases, Regional Branch in Rabka Zdrój, 34-700 Rabka-Zdroj, Poland; wozniak.m.1986@gmail.com (M.W.); zdoniec@interia.pl (Z.D.); 5 Department of Diagnostic Imaging, Mother and Child Institute, 01-211 Warsaw, Poland; kwasniewiczp@gmail.com; 6 Pediatric Department of Respiratory Tract Disorders, Lung Diseases and Rehabilitation Center, 91-520 Łódź, Poland; sonokasia@dot.pl; 7 Department and Clinic of Internal Medicine, Connective Tissue Diseases and Geriatrics, Medical University of Gdańsk, 80-210 Gdansk, Poland; 8 Department of Pediatrics, Hematology and Oncology, Medical University of Gdansk, 80-210 Gdansk, Poland; kwojtek@gumed.edu.pl

**Keywords:** paediatric pneumonia, bronchiolitis, point-of-care ultrasound, LUS

## Abstract

This evidence-based consensus aims to establish the role of point-of-care lung ultrasound in the management of pneumonia and bronchiolitis in paediatric patients. A panel of thirteen experts form five Polish tertiary pediatric centres was involved in the development of this document. The literature search was done in PubMed database. Statements were established based on a review of full-text articles published in English up to December 2019. The development of this consensus was conducted according to the GRADE**** (Grading of Recommendations, Assessment, Development and Evaluations)—adopted and Delphi method. Initially, 22 proposed statements were debated over 3 rounds of on-line discussion and anonymous voting sessions. A total of 17 statements were agreed upon, including four statements referring to general issues, nine referring to pneumonia and four to bronchiolitis. For five statements experts did not achieve an agreement. The evidence supporting each statement was evaluated to assess the strength of each statement. Overall, eight statements were rated strong, five statements moderate, and four statements weak. For each statement, experts provided their comments based on the literature review and their own experience. This consensus is the first to establish the role of lung ultrasound in the diagnosis and management of pneumonia and bronchiolitis in children as an evidence-based method of imaging.

## 1. Introduction

Although the diagnosis of respiratory tract infections, including pneumonia and bronchiolitis, is based on clinical data, in some cases, performance of imaging modalities is necessary [1]. Chest X-ray (CXR) is the most commonly performed test. However, it has significant limitations such as patient exposure to ionising radiation, relatively low sensitivity in detecting pulmonary inflammatory lesions, low negative predictive value (NPV), and interpretation discrepancies among specialists [2]. In comparison, the diagnostic performance of chest computed tomography (chest CT) is much better, but as it exposes patients to a high radiation dose, the benefit–risk balance does not allow for its routine use in children [3]. The radiation exposure of CT could be avoided using MRI. Modern technology has enabled high-quality three-dimensional lung imaging with this tool, which proved especially promising for the detection of complicated pneumonia. However, it is still too expensive, and like CT may demand sedation or anesthesia in young, uncooperative children, demands long training and thus is not widely available even in tertiary centers [4].

Lung ultrasound (LUS) is an alternative tool unburdened with the above-mentioned limitations. Numerous advantages of LUS include continuous bedside availability, short performing time, no radiation risk, smaller dependence on a patient’s movements (including crying), relatively low costs, and an encouraging learning curve [5]. It can also be repeated multiple times if needed, and therefore can be used for treatment monitoring [6].

In emergency medicine, sonographers are equipped with standardised LUS protocols enabling rapid patient assessment [7,8]. For adult patients, both Polish recommendations on LUS application in internal medicine [9,10], and international evidence-based recommendations on the point of care lung ultrasound are followed. Few recommendations regarding the use of LUS in neonatal and paediatric diseases are outlined in the latter [11]. Nevertheless, the role of LUS in a paediatric population is not firmly established yet.

The purpose of this consensus is to define the position of point-of-care LUS in the management of pneumonia and bronchiolitis in children.

## 2. Materials and Methods

### 2.1. Expert Panel Selection

An expert panel of specialists from five different Polish tertiary paediatric centres was selected. Experts (paediatricians, peadiatric pulmonologists and radiologists) who had published articles regarding LUS in the past 10 years and/or have at least 4 years of experience in performing LUS, with at least 300 examinations per year, were chosen.

### 2.2. Literature Search

**Figure 1:**
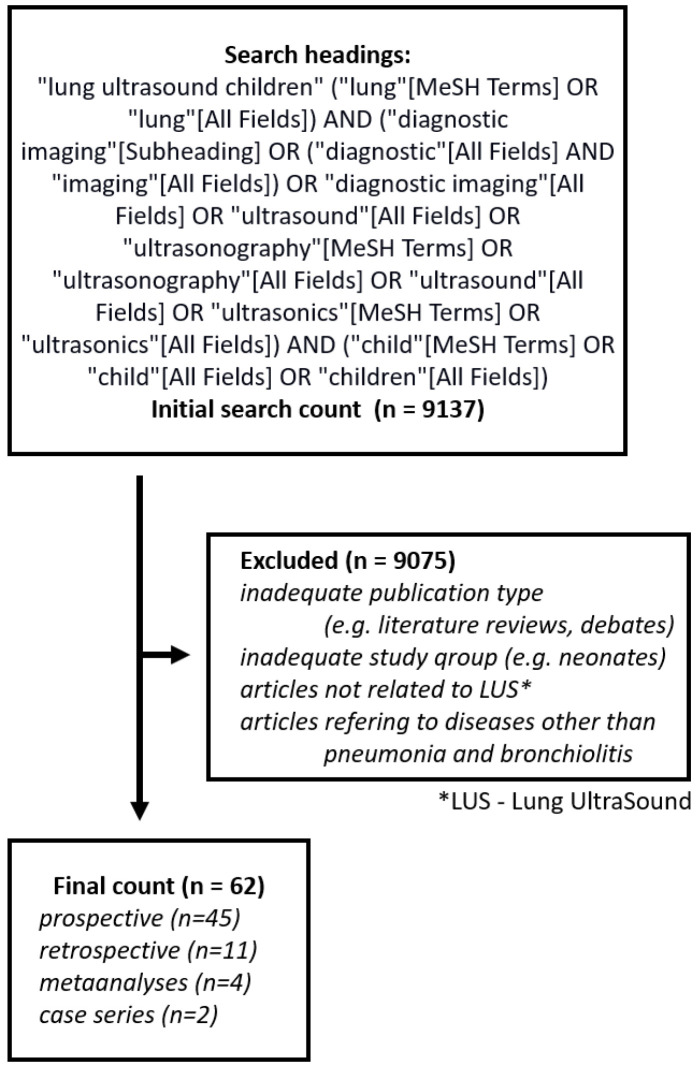
PRISMA (Preferred Reporting Items for Systematic Reviews and Meta-Analyses) flow diagram.

The literature search was conducted in PubMed database and included articles published prior to January 2020. The search included headings: “lung ultrasound children”. The literature search and review process is presented in Figure 1. The database containing all the publications was created using the free on-line program Zotero available at www.zotero.org. The articles limited to perinatal, neonatal, and adult patients were excluded except for articles which included both children and young adults (up to 21 years of age). For further evaluation, only English language full-text original papers were chosen including meta-analyses, case series, but not case reports. Since there is a very limited number of publications focusing on the use of LUS in children with pneumothorax, foreign body aspiration, cystic fibrosis, atelectasis, chest tumours, congenital abnormalities of the respiratory tract and undergoing cardiac surgery, the expert panel has decided to narrow the scope of the analysis to pneumonia and bronchiolitis.

### 2.3. Statement Development

Experts submitted proposals for possible statements while paying special attention to the following: examination technique, diagnostic criteria for pneumonia and bronchiolitis, the role of LUS in the management of these diseases, comparison of LUS to other imaging methods, and finally, the role of LUS in the follow-up of the patients. Each statement was independently assessed in terms of the degree of agreement/disagreement between experts.

**Figure 2:**
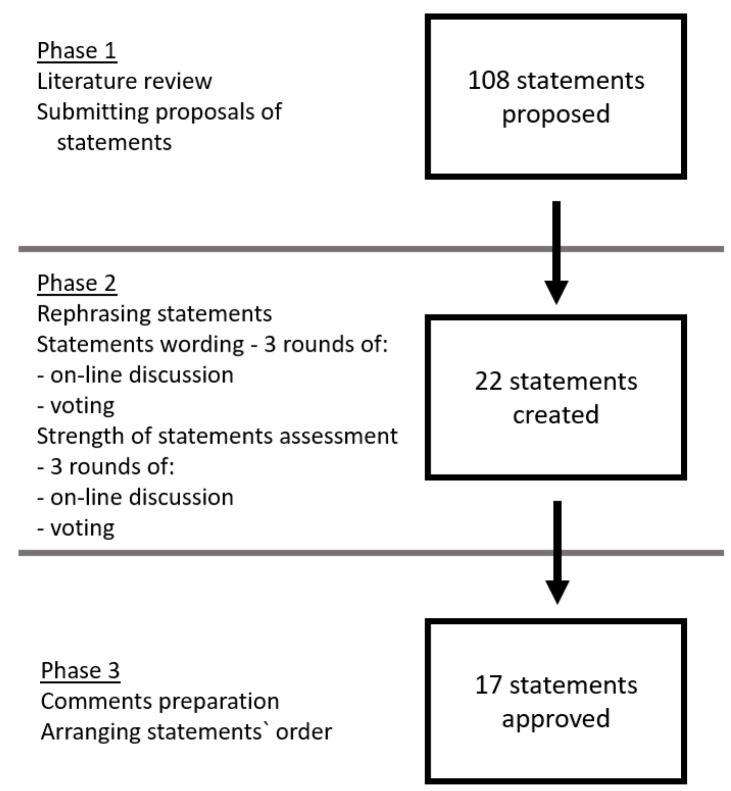
The process of statements’ development and strength assessment.

In compliance with Delphi method, the experts shared their opinions about the statements in 3 on-line discussion rounds, each round being followed by anonymous voting [12]. If a statement received ≥80% positive votes or >50% negative votes, the statement was approved and incorporated in the consensus. If the voting result was still indeterminate after third Delphi round, the statement was rejected (Table 1). The process of statements development and strength assessment is presented in Figure 2.

### 2.4. Strength of Statements

The assessment of statements’ strength was based on the quality of evidence and parameters including sensitivity, specificity, positive and negative predictive values, and positive and negative likelihood ratios, following GRADE-adopted methodology [13]. Quality of evidence was assessed following the Tool for Quality Assessment of Diagnostic Accuracy Studies, which included such variables as publication type, study group size and homogeneity, inclusion and exclusion criteria, a diagnostic method used as a reference and criteria for establishing the final diagnosis [14]. Similarly to statement development, the strength of evidence for each formulated statement was rated as either high (A), moderate (B), low or very low (C) (Table 2 and Table 3). The strength assessment group consisted of eight experts.

### 2.5. Comments

For each statement, the authors provided comments based not only on the literature review, but also on their own experience. Experts paid special attention to addressing practical issues regarding LUS performance. Basic definitions of LUS findings are presented in Table 4 [15].

### 2.6. Statements

Experts’ shared opinion preceding statements: Interpretation of LUS should be performed within a clinical context [16,17,18,19,20,21,22,23,24,25,26,27,28].

## 3. General Statements

1. A linear transducer is the most commonly used transducer for LUS examination of a suspected lower respiratory tract infection (LRTI) in children (A1) [29,30,31,32,33,34].
  Comments:
  a. Other transducers used for LUS examination in children include convex, microconvex, and sector [24,29,30,31,35,36,37,38,39].
  b. The appropriate transducer must balance acquiring the best possible image quality with the maintenance of adequate ultrasound wave penetration. The examining conditions may be affected by:
    - The distance between the transducer and the pleural line—when the distance is greater, e.g., in children with excessive adipose tissue, or when using the transabdominal approach, low-frequency transducers may be necessary [40,41,42];
    - Lesion size—in case of abnormalities involving a large area of the lung parenchyma (e.g., large consolidations) or massive pleural effusion, transducers with a wide field of view;
    - Deeper penetration may prove necessary [33].
  c. Differentiation between B-lines and other vertical artefacts (I- and Z-lines) may require the use of a convex or microconvex transducer.
2. The entire available lung surface should be examined in children with suspected LRTI (A1) [31,35,42,43,44,45,46,47].
  Comments:
  a. The recommended examination technique includes the assessment of the entire available lung surface, i.e., anterior, lateral and posterior surface of both lungs [6,16,19,23,25,28,34,42,43,44,45,48,49,50,51,52,53,54,55]. The examined area may be limited to anterior and lateral surfaces in the case of patients who are:
    - Hospitalised in the intensive care unit due to the risk posed by changing their position;
    - In life-threatening conditions, examined following quick-assessment protocols, such as EFAST, BLUE, PEA, RADIUS.
  Should examining the entire available lung surface prove impossible (due to patients’ anxiety), such information must be included in the examination result.
  b. The transthoracic examination requires placing the transducer both in sagittal and transverse planes [43,45].
  c. Supraclavicular projection enables the assessment of the lung apices [45,47].
  d. Some patients may benefit from extending the examination by:
    - Transabdominal projection, which allows for lung base evaluation [40,46];
    - Assessing the mediastinal pleura using the heart as an acoustic window;
    - Mediastinum assessment [47].
3. Diagnostic value of LUS in children with suspected LRTI to a limited extent depends on the sonographer’s experience (B1) [16,27,28,47,56,57].
  Comments:
  - LUS has high sensitivity and specificity in detecting community-acquired pneumonia (CAP) in children, even when performed by sonographers with limited experience [16,18,29,34,39,56,58,59].
  - Sonographers, who are novices in diagnosing CAP, can acquire high concordance with experienced sonographers relatively quickly [56].
  - Many publications agree that the interpretation of LUS findings among doctors is accurate regardless of their specialty [27,28,29,52,54,57,60,61,62].
4. LUS has high diagnostic value in assessing the presence of fluid in pleural cavities (A1) [16,18,24,26,32,35,36,38,40,44,48,60,63,64].
  Comments:
  - Publications comparing LUS with CXR or chest CT proved that ultrasound provides visualization of small-volume pleural fluid, not detectable with other imaging methods [16,24,26,32,35,36,38,44,49,63,64].
  - In case of small-volume fluid detection with LUS, it is crucial to evaluate the fluid’s clinical significance.
  - LUS enables visualization of small-volume pleural fluid directly adjacent to the area of consolidation.
  - LUS enables the characteristics of pleural fluid to be assessed (anechoic, containing fibrin strains, septations, loculations, pleural adhesions) [32,36].
  - To standardise the model result of LUS in case of pleural effusion, we suggest including the following information: the patient’s position during the examination, the precise location (including the maximal height of the fluid, its maximal depth with determining the intercostal space and body line) as well as the characteristics of the fluid.

### 3.1. Pneumonia

5. LUS is useful for diagnosing CAP in children (A1) [6,16,19,20,22,24,25,26,27,29,30,33,35,36,37,38,39,40,41,43,44,45,48,49,51,55,57,58,60,65,66,67,68].
  Comments:
  - In children suspected of CAP, LUS is more effective in diagnosing the disease than the physical examination alone or physical examination combined with complete blood count and CXR [16,20,29,46].
6. LUS has at least equal diagnostic value to CXR in detecting CAP in children (A1) [6,16,19,26,30,35,37,39,41,44,45,48,51,55,58,60].
  Comments:
  - In most patients with suspected pneumonia, who had normal CXR results and LUS findings suggesting pneumonia, the clinical course and chest CT confirmed pneumonia [6,16,19,30,32,38,44,45,49].
  - LUS enables the reduction in CXR use for CAP diagnosis in children without lowering diagnostic accuracy and safety [25,29,39,45,50,58,66,67].
  - We suggest LUS as the first-choice method in paediatric patients suspected of CAP who require diagnostic imaging [65,67]. However, in case of discrepancy between LUS and clinical findings, CXR performance should be considered.
  - Some patients will benefit from having both CXR and LUS performed, as these two imaging methods should be regarded as complementary [40].
7. Normal LUS results in children with suspected LRTI significantly reduce the probability of diagnosing CAP (A1) [16,24,25,26,33,41,49,51,55,60,67].
  Comments:
  - In most children presenting with symptoms of respiratory tract disease, who had normal LUS results, a final diagnosis other than pneumonia was established [6,19,26,37,41,60].
  - Despite high sensitivity of LUS in diagnosing pneumonia, some lesions remain undetectable (false-negative result) if the lesion:
    - Does not adhere to the pleura (e.g., in perihilar areas) [26,29,33,35,37,40,67]; or
    - Is located in an area inaccessible for the ultrasound (e.g., retroscapular areas) [26,33,40].
8. Consolidation is the most commonly reported LUS finding in children with pneumonia (A1) [6,20,22,24,26,29,30,32,33,35,37,39,41,42,46,51,58,60,67,69].
  Comments:
  - Should a consolidation be detected, it is crucial to measure it in at least two, optimally three, dimensions [67].
  - Other LUS findings consistent with pneumonia include: air bronchogram within the area of consolidation, B-line artifacts, interstitial syndrome, pleural effusion, irregular pleural line, and superficial fluid alveologram [19,30,32,37,38,39,41,48,51,55,67].
9. LUS is more sensitive in detecting consolidations than CXR (A1) [16,28,33,39,44,48,49,51,58].
  Comments:
  - Discrepancies in detecting lung consolidations between CXR and LUS are mainly related to the ability of the ultrasound to identify small consolidations (≤10 mm) [16,33,39].
10. Assessment of the vascular pattern of the consolidation may improve the diagnostic value of LUS in children with suspected LRTI (C1) [31,36,38].
  Comments:
  - Doppler imaging can differentiate fluid bronchogram within atelectasis from fluid within bronchial vessels [38,57].
  - In cases suspected of complicated pneumonia, Doppler imaging can identify hypoperfused lung parenchyma [31,36].
  - An abnormal vascular pattern may be indicative of other aetiology of the lesions (tuberucolous, fungal or non-infectious).
  - Vascular pattern assessment may be hindered in cases of examining an anxious child, small consolidations or lesions located in the pericardiac area (due to cardiac pulse).
11. LUS does not determine the aetiology of CAP in children (C1) [27,42].
  Comments:
  - Like other imaging techniques, LUS cannot indicate the aetiologic factor. However, regarding pathophysiology and the whole clinical picture, LUS findings can be suggestive of certain groups of pathogens. Features suggestive of viral or atypical aetiology include bilateral consolidations, which are usually smaller compared to the ones observed in bacterial pneumonia, as well as more frequent presence of B-lines forming interstitial syndromes [16,27,58].
12. LUS is useful in monitoring the course of pneumonia in children (B1) [6,37,38,42,51,67,70].
  Comments:
  - In patients with a good response to the treatment of pneumonia, regression of LUS findings is observed, including consolidation size reduction, gradual re-aeration of the lung parenchyma, decrease in the number of B-lines, and in the volume of pleural fluid [6,38,57,71].
  - LUS findings regression correlates with the normalisation of the acute phase reactants as well as a clinical improvement [6,37,51,67,71].
  - Progression or no regression of LUS findings correlates with no clinical improvement and poor response to the treatment [6,38,51,71].
  - In patients with pneumonia, LUS monitoring enables early detection of complications [38].
  - It is worth performing an additional control LUS examination 1-2 months after the treatment has been completed. The knowledge of residual findings can facilitate correct interpretation of LUS, should the patient be suspected of a next episode of LRTI [21].
13. LUS is useful in diagnosing complications of pneumonia in children (B1) [31,36,40,46,57].
  Comments:
  - Massive consolidations (>50mm) and fluid bronchogram that present in the peripheral areas of consolidations may serve as prognostic factors for complicated pneumonia [36,40,57].
  - LUS a has similar diagnostic accuracy as chest CT in regards detecting areas of necrosis within consolidated lung parenchyma [31,36,40,46].
  - LUS accuracy of lung abscess detection depends mainly on localisation of the lesion.
  - LUS seems to be useful in diagnosing pneumothorax in children, though it does not allow air volume assessment, and it cannot aid in the choice of the treatment method.

### 3.2. Bronchiolitis

Bronchiolitis is per se a clinical diagnosis and therefore there is no need for routine imaging evaluation [72,73]. However, in some patients, who are at risk of requiring intensive therapy, in whom complications or other diagnosis is being considered, imaging modalities may prove helpful. The statements below relate to the latter situation.

14. LUS is useful in bronchiolitis diagnosis (B1) [23,52,53,54,63,64].
  Comments:
  - LUS findings consistent with bronchiolitis are usually spread bilaterally and include subpleural consolidations, multiple B-lines, pleural line abnormalities and minimal pleural effusion [23,28,52,53,54,63,74].
15. LUS has a diagnostic value equal or greater than CXR in bronchiolitis diagnosis (C1) [23,63,64].
  Comments:
  - We suggest LUS as the first-choice method in patients with bronchiolitis who require diagnostic imaging.

As LUS is a relatively new method and has certain limitations (as described above), in case of equivocal results, it may be reasonable to perform both—LUS and CXR. LUS is not only safer (no radiation risk), but also allows assessment of the severity of bronchiolitis, as mentioned below (statement 16, comments a, b and c).

16. LUS is useful in assessing the severity of bronchiolitis (B1) [23,34,52,53,54,64].
  Comments:
  - In patients with bronchiolitis, a normal LUS image is usually associated with a mild course of infection [53,54,64].
  - There is a good correlation between ultrasonographic and clinical findings in bronchiolitis [53,54,64].
  - The Bronchiolitis Ultrasound Score (BUS) is a specific tool, which can be used for the identification of infants with bronchiolitis and with an increased risk of needing supplementary oxygen and/or respiratory support. It can also be used to predict hospital admission [23,52,53,54].
17. LUS is useful in monitoring patients with bronchiolitis (C1) [54,64].
  Comments:
  - The regression in the size and number of consolidations and in the size of the interstitial syndrome area have been associated with an improvement in the clinical course of bronchiolitis [54,64].

The statements, grouped by their strength, are presented in Table 5.

## 4. Discussion and Conclusions

To the best of the authors’ knowledge, this is the first evidence-based document substantiating the role of LUS in the management of pneumonia and bronchiolitis in children. Volpicelli et al. published international recommendations, which included only three statements concerning the paediatric population [11]. Our consensus complies with two of the statements entirely: 1. “LUS is a clinically useful tool in children with suspected pneumonia” and 2. “LUS has at least the same diagnostic value as chest X-ray in diagnosing” this disease entity. In this document, we did not address the third statement: “The ultrasound signs of lung and pleural diseases described in adults are also found in paediatric patients” as we did not analyse data from adults. Volpicelli also states that “if pneumonia is suspected, positive LUS excludes the need to perform CXR”, which supports our suggestion to treat LUS as the first-choice method in paediatric patients suspected of CAP who require diagnostic imaging (statement 6, comment c).

There are some limitations to our consensus. Firstly, the literature search was performed in only one database and included only articles published in English. Secondly, even though the literature search was wide, there were few documents concerning LUS in children. Moreover, due to constrained data regarding the use of LUS in many respiratory tract diseases in children, the spectrum of this consensus had to be limited to pneumonia and bronchiolitis. Finally, although all the statements are firmly based on evidence research, the comments are based both on the literature review and the authors’ personal experiences. Despite all the above-mentioned limitations, this consensus establishes the role of LUS in the management of pneumonia and bronchiolitis in children. It gathers the opinions of experts in different fields of respiratory tract diseases ranging from radiologists, paediatricians, to paediatric pulmonologists. Moreover, it addresses many practical issues regarding the performance technique and the result preparation process of LUS and, therefore, may provide guidance for sonographers of different levels of experience. Finally, this document also addresses all the physicians who do not perform LUS themselves but utilize the results obtained by LUS. In this case, it offers guidance on when to order LUS, its capabilities and limitations, and how to interpret the results of this relatively new imaging tool. Given the possibility of acquiring clinically significant information with the use of LUS, the authors expect that in the next few years this modality will be included in the new guidelines and recommendations for the diagnosis and management of respiratory tract infections in children.

The consensus will be updated every three years as new relevant reports in the literature will emerge.

Articles included in the literature review, but not cited before: [75,76].

## Figures and Tables

**Table 1:** Degree of experts’ agreement, Delphi method.

Experts’ Opinion	% Positive Votes
Agreed for—1	≥80%
Agreed against—2	≤50%
Indeterminate—rejected	51–79%

**Table 2:** GRADE-adopted classification.

Level of Evidence	Criteria for Quality of Evidence
A (high)	Data come from many meta-analyses, and/or it is unlikely that further research will change the credibility of effectiveness or accuracy of the method.
B (moderate)	Data come from individual large non-randomized trials (meta-analysis, prospective cohort study), and/or further testing may have a significant impact on the credibility of effectiveness or accuracy of the method.
C (low or very low)	Agreed expert opinion and/or data from small studies, retrospective studies, registers, case series, or case reports, and/or it is very likely that further testing will have an important impact on the credibility of effectiveness or accuracy of the method. Very low in case any estimation of the effects or accuracy of the method is very uncertain.

**Table 3:** Strength of statements.

Expert’s Opinion	Level of Evidence	Strength of Statement	Strength of Statement—Practical Implications
1	A	A1	Strong statement; the given statement should be widely followed, as long as there are no major obstacles.
1	B	B1	Strong statement, but with less degree of certainty; probably right in most individual cases.
1	C	C1	The average (moderate) strength of statement; the statement may change after obtaining more reliable data; probably right.
2	A	A2	The average (moderate) strength of statement; the decision on its adoption is a matter of choice and may depend on local and individual conditions; intervention does not have to be used.
2	B	B2	Weak statement; alternative conduct can be just as good or better.
2	C	C2	Weak statement; alternative conduct is probably equally acceptable.

**Table 4:** Basic definitions of lung ultrasound (LUS) findings.

LUS Finding	Definition
A-lines (A-line artefacts)	Repetition of the pleural line at a standardized distance equal to the skin–pleural line distance.
B-lines (B-line artefacts)	Comet-tail artefacts that arise from the pleural line and move simultaneously with the breathing cycle. The other optional 4 criteria are: screen-long, well-defined, erasing A-lines, and hyperechoic.
Consolidation	Hypoechoic, subpleural tissue-like area, caused by fluid displacing alveolar air. In case of a large consolidation, the appearance is characteristically liver-like. Usually, a consolidation has blurred margins and the following associated features: – The loss of pleural line echogenicity over the area of consolidation and the absence of A-lines within the area. – Comet-tail artefacts arising from the deep edge of the consolidation. – B-lines surrounding the area of consolidation. – An air bronchogram—observed as multiple hyperechoic specks or branching tree-like structure within the area of consolidation: a) Dynamic—moving simultaneously with the breathing cycle; or b) Static. – A fluid bronchogram—an anechoic or hypoechoic branched tubular structure along the airways, within the area of consolidation. – Vascular pattern in color Doppler option—observed as branching tree-like structures with blood flow.
I-lines, Z-lines (I- and Z-line artefacts)	Short vertical hyperechoic artefacts arising from the pleural line, not reaching the distal edge of the screen.
Interstitial syndrome	≥3 B-lines visible in the longitudinal plane between two ribs.

**Table 5:** The statements grouped by their strength.

Strength of Statement *	Statement	
A1	1.	A linear transducer is the most commonly used transducer for LUS examination of a suspected lower respiratory tract infection (LRTI) in children.
	2.	The entire available lung surface should be examined in children with suspected LRTI.
	4.	LUS has high diagnostic value in assessing the presence of fluid in pleural cavities.
	5.	LUS is useful for diagnosing community-acquired pneumonia (CAP) in children.
	6.	LUS has at least equal diagnostic value to chest X-ray (CXR) in detecting CAP in children.
	7.	Normal LUS results in children with suspected LRTI significantly reduce the probability of diagnosing CAP.
	8.	Consolidation is the most commonly reported LUS finding in children with pneumonia.
	9.	LUS is more sensitive in detecting consolidations than CXR.
B1	3.	Diagnostic value of LUS in children with suspected LRTI to a limited extent depends on the sonographer’s experience.
	12.	LUS is useful in monitoring the course of pneumonia in children.
	13.	LUS is useful in diagnosing complications of pneumonia in children.
	14.	LUS is useful in bronchiolitis diagnosis.
	16.	LUS is useful in assessing the severity of bronchiolitis.
C1	10.	Assessment of the vascular pattern of the consolidation may improve the diagnostic value of LUS in children with suspected LRTI.
	11.	LUS does not determine the aetiology of CAP in children.
	15.	LUS has a diagnostic value equal or greater than CXR in bronchiolitis diagnosis.
	17.	LUS is useful in monitoring patients with bronchiolitis.

* No statements were ranked A2, B2 or C2.
